# GBDT-IL: Incremental Learning of Gradient Boosting Decision Trees to Detect Botnets in Internet of Things

**DOI:** 10.3390/s24072083

**Published:** 2024-03-25

**Authors:** Ruidong Chen, Tianci Dai, Yanfeng Zhang, Yukun Zhu, Xin Liu, Erfan Zhao

**Affiliations:** 1 Institute for Cyber Security, School of Computer Science and Engineering, University of Electronic Science and Technology of China (UESTC), Chengdu 611731, China; crdchen@uestc.edu.cn (R.C.); dtc1234564@163.com (T.D.); n3bul2@feishu.uestc.cn (Y.Z.); zhaoerfan@gmail.com (E.Z.); 2 Intelligent Policing Key Laboratory of Sichuan Province, Sichuan Police College, Luzhou 646000, China; 3 School of Information Science and Engineering, Lanzhou University, Lanzhou 730000, China; bird@lzu.edu.cn

**Keywords:** botnets, internet of things, feature dimensionality reduction, concept drift

## Abstract

The rapid development of the Internet of Things (IoT) has brought many conveniences to our daily life. However, it has also introduced various security risks that need to be addressed. The proliferation of IoT botnets is one of these risks. Most of researchers have had some success in IoT botnet detection using artificial intelligence (AI). However, they have not considered the impact of dynamic network data streams on the models in real-world environments. Over time, existing detection models struggle to cope with evolving botnets. To address this challenge, we propose an incremental learning approach based on Gradient Boosting Decision Trees (GBDT), called GBDT-IL, for detecting botnet traffic in IoT environments. It improves the robustness of the framework by adapting to dynamic IoT data using incremental learning. Additionally, it incorporates an enhanced Fisher Score feature selection algorithm, which enables the model to achieve a high accuracy even with a smaller set of optimal features, thereby reducing the system resources required for model training. To evaluate the effectiveness of our approach, we conducted experiments on the BoT-IoT, N-BaIoT, MedBIoT, and MQTTSet datasets. We compared our method with similar feature selection algorithms and existing concept drift detection algorithms. The experimental results demonstrated that our method achieved an average accuracy of 99.81% using only 25 features, outperforming similar feature selection algorithms. Furthermore, our method achieved an average accuracy of 96.88% in the presence of different types of drifting data, which is 2.98% higher than the best available concept drift detection algorithms, while maintaining a low average false positive rate of 3.02%.

## 1. Introduction

The rapid development of IoT has brought many conveniences to our daily life. However, it has also introduced various security risks that need to be addressed. According to estimates, by 2025, IoT and related applications are projected to generate a potential economic impact ranging from USD 3.9 trillion to USD 11.1 trillion annually [1]. IoT devices can evolve into smart objects by leveraging their core technologies, including communication technologies, ubiquitous computing, embedded devices, internet protocols, sensor networks, and AI-based applications [2]. This transformation has brought positive impacts to various aspects of life, such as transportation, healthcare, energy management, and autonomous driving. However, the openness and diversity of IoT networks, along with the inherent security vulnerabilities of IoT devices, have made them an ideal breeding ground for the proliferation of botnets. A famous example is the Mirai botnet, which was first discovered by white hat security agency MalwareMustDie in August 2016, and many variants and imitators of Mirai have become the vector for the most powerful DDos attacks in history [3]. Moreover, IoT botnets typically consist of a massive number of compromised devices, and the attack traffic they generate often exceeds the threshold of website crashes by several orders of magnitude. This poses significant challenges to existing security defense mechanisms. Consequently, there is a growing demand for new approaches to detect botnet behaviors within IoT device networks.

To cope with the problem of botnets, researchers have proposed many detection algorithms, primarily including host-based and network traffic-based detection methods. Currently, with the rapid development of AI technology, machine learning (ML) and deep learning (DL) methods are widely used in botnet detection. The basic approach is to leverage network traffic features and build models for identifying botnet behaviors, enabling the differentiation between benign and anomalous traffic [4]. However, DL- or ML-based botnet detection systems often demand substantial computational resources. This poses a challenge for their deployment in IoT environments due to the high heterogeneity, limited storage, and computing capabilities of IoT devices [5]. Moreover, IoT data are typically dynamic and non-stationary, with data distributions changing over time, leading to the phenomenon of concept drift. When detecting botnet activities in the presence of concept drift, the detection models often suffer from performance degradation [6].

In order to solve the above challenges, we designed a botnet detection model called GBDT-IL. This model is an enhanced version of the GBDT model. It incorporates incremental learning to adapt to concept drift in the data and combines it with pruning operations to optimize the model further and reduce computational resource usage. In addition, it employs an improved Fisher Score feature selection algorithm for feature dimensionality reduction of the dataset features, which further reduces the system resources required for model training. To verify the performance of the model, three different conceptual drift forms were constructed to test the model. The experimental results showed that the proposed botnet detection model of GBDT-IL has good classification performance in dealing with different situations and different degrees of concept drift. The main contributions of our paper include the following three points:

1 We propose an improved feature selection method with Fisher Score, which filters out the best features based on the score and eliminates most irrelevant features. While ensuring high accuracy of the model, it further reduces the system resources required for model training, making the detection model applicable to resource-poor IoT devices.
2 We propose a gradient boosting decision-tree-improvement-based anti-conceptual drift algorithm, GBDT-IL (Gradient Boosting Decision Tree-incremental learning), which adapts to emerging data samples in the data stream by the incremental learning method. Also considering the overfitting due to the redundancy of the incremental learning process, the tree-pruning process is added to improve the model performance.
3 We validate our method on four commonly used IoT datasets as well as their constructed drift datasets. The experimental results show that the improved feature selection method with Fisher Score outperforms and significantly reduces the training time of the model compared to existing feature selection methods. In addition, GBDT-IL is able to improve the model accuracy by more than 20% compared to traditional machine learning algorithms, and it also performs better than existing concept drift-resistant algorithms.

The remainder of this paper is arranged as follows: Section 2 describes the related works on botnet detection and concept drift handling. In Section 3, our proposed GBDT-IL is illustrated in detail. The analysis of the experimental results of GBDT-IL is presented in Section 4. Section 5 is a summary of the thesis and an outlook for the future.

## 2. Related Works

This section reviews related works on botnet detection methods and anti-conceptual drift techniques.

### 2.1. Botnet Detection Methods

With the rapid development of IoT, the security issues caused by various IoT botnets have drawn increasing attention from individuals across different sectors of society. Scholars from both domestic and international communities have proposed numerous methods for detecting botnet activities.

Back in 2012, Leyla Bilge et al. [7] proposed a wide- and large-scale traditional botnet detection system, and they used various machine learning algorithms, such as decision trees, support vector machines, and random forests, to conduct experiments on botnet detection. The best result among the three algorithms in terms of accuracy is random forest, which can reach an accuracy rate of 87.8% and a false alarm rate of about 20%. It can be seen that their proposed method has more room for improvement in all aspects.

In 2017, Ruidong Chen et al. [8] proposed a real-time network-intrusion detection system: a supervised machine learning approach to detect botnets in a high-speed network environment. The detection framework is first constructed through the PF_RING high-speed packet processing framework for the dynamic extraction of network traffic features, and then a machine learning model is used to extract session features. Finally, through experiments, it was concluded that the accuracy of the method can reach 94%.

In 2018, in the approach of Nour Moustafa et al. [9], a set of traffic characteristics based on statistical information is proposed through an in-depth analysis of the protocols commonly used in IoT environments, especially MQTT, DNS, and HTTP protocols. Meanwhile, based on three techniques, namely, decision tree, naive Bayesian, and artificial neural network, AdaBoost integrated learning is used to make improvements to the overall performance of the system in terms of detection accuracy and processing time. The study showed that the proposed integration method outperformed the existing techniques in the integration method on the data sources of DNS and HTTP protocols. In the same year, Sajad Homayoun et al. [10] used a deep learning-based botnet traffic analyzer, BoTShark, which is independent of deep packet inspection techniques and uses only network data streams, effectively avoiding the limitation of not being able to handle encrypted payloads. The authors used the ISCX 2014 botnet data as the experimental dataset and then detected the malicious traffic in the dataset by auto-encoder and convolutional neural network, respectively, which yielded 91% true positives and 92% true positives, and 13% false positives and 15% false positives for the auto-encoder and convolutional neural network, respectively.

In 2020, SI Popoola et al. [11] used the encoding phase of the long and short-term memory auto-encoder to reduce the feature dimensionality of large-scale IoT network traffic data. Also, to correctly classify network traffic samples, they used a deep bidirectional long and short-term memory (BLSTM) to analyze the long-term interrelated changes in the low-dimensional feature set generated by LAE. Extensive experiments on the BoT-IoT dataset validated the effectiveness of the proposed hybrid DL approach. The results showed that LAE significantly reduced the memory space required for storing large-scale network traffic data, outperforming existing feature reduction methods. Despite the significant reduction in feature size, the deep BLSTM model showed robustness to model underfitting and overfitting. A good generalization capability was also achieved in binary and multiclass classification scenarios.

In 2021, Javed Asharf et al. [12] proposed a new statistical learning-based botnet detection framework-IoTBoT-IDS. By applying statistical learning-based techniques using the Beta Mixture Model (BMM) and Correntropy model to capture the normal IoT network behavior, any deviations from the normal behavior are detected as anomalous events. The evaluation results showed that IoTBoT-IDS can effectively identify various types of botnets with an average detection accuracy of 99.2%.

However, the aforementioned botnet detection methods have not taken into account the impact of concept drift on the detection models. Considering the rapid development of IoT devices, concept drift is bound to become a significant factor affecting the performance of detection models. Therefore, it is crucial to incorporate concept drift-resistant algorithms into detection models.

### 2.2. Concept Drift Detection

With the rapid development of IoT, the amount of data generated by it also increases in an explosive scale. Some phenomena in the data streams lead to the inability of some traditional classification algorithms to meet their classification needs, and the concept drift problem in the data streams has received a lot of focused attention from scholars domestically and internationally.

Back in 2013, Ditzler et al. [13] used an improved algorithm based on the incremental learning algorithm Learn++.NSE for the concept drift problem present in data streams. They experimented with the algorithm using several datasets where concept drift occurred, and the final results showed that in certain non-smooth environments, such as Gaussian distribution drift, the algorithm outperformed the traditional machine learning algorithm.

In 2015, D Brzezinski et al. [14] proposed a new algorithm, called OnlineAUE, by studying the problem of constructing integrated classifiers for accurate classification from data streams where conceptual drift occurs, which extends AWE by using online component classifiers and updating them according to the current distribution. Additional modifications to the weighting function solve the unwanted classifier exclusion problem that occurs in AWE. Experiments conducted with several datasets where conceptual drift occurs showed that OnlineAUE classification accuracy was significantly higher than its predecessor AWE, but there is more room for improvement in its accuracy.

In 2019, Frias-Blanco et al. [15] proposed a new method to monitor the performance metrics measured during online learning and to trigger a drift signal when significant changes are detected. The authors proposed two main methods in their work. The first method, called HDDM-W-Test, utilizes a moving average approach and is particularly effective in detecting mutations in data. On the other hand, the second method, known as HDDM-A-Test, addresses gradual changes by employing a weighted moving average technique. The simplicity of the proposed methods as well as their computational efficiency make them very favorable, and finally, the performance of these methods on synthetic and real data is evaluated using a plain Bayesian classifier and perceptron.

In 2021, Hanli Qiao et al. [16] proposed a dynamic sliding window method based on residual subspace projection to study how concept drift analysis affects the performance of cyber-attack detection in IoT scenarios. They designed the sub-dataset of Bot-IoT to ensure that concept drift occurs for finalizing the experiments. The detection accuracy was improved by 15% to 26% compared to the classification model without concept drift analysis, where the classification algorithms use CNN and LSTM, respectively. They also obtained excellent performance results by comparing the confusion matrix when performing concept drift analysis.

In 2022, in order to address the problem of data drift and conceptual drift due to the fact that IoT is a highly dynamic and heterogeneous environment, a drift detection technique was proposed by Omar Abdul et al. [17]. This technique utilizes Principal Component Analysis (PCA) to examine changes in feature variance within intrusion detection data streams. They also discussed an online anomaly detection technique capable of identifying outliers different from historical and temporally proximate data points. To tackle these drifts, they explored an online Deep Neural Network (DNN) that dynamically adjusts the size of hidden layers based on the Hedge weighted mechanism. This enables the model to steadily learn and adapt with the arrival of new intrusion data. Experimental results conducted on an IoT-based intrusion detection dataset indicated that, compared to widely-used static DNN models for intrusion detection, their solution exhibited stable performance on both training and testing data.

In 2023, Mohammed Amin et al. [18] designed a statistical bias detection method to ensure the security of the Industrial Internet of Things (IIoT). The approach aims to detect any changes in data patterns and employs a machine learning classifier to combat newly developed malicious software samples. The method involves sampling a portion of new data in each batch, and the model’s performance is assessed using the F1 score. If the F1 score falls below an acceptable threshold or the statistical model suggests retraining, the classifier automatically triggers a process to re-label data and retrain the model. This allows the model to maintain performance while adapting to modifications in data distribution. The proposed method was experimentally validated in drift scenarios using the publicly available IoT-23 dataset. The experimental results demonstrated a high accuracy rate of 95.2% and an F1 score of 94%, showcasing the method’s success and ease of adoption. Adel Abusitta et al. [19] proposed a deep learning-based method for anomaly detection in Internet of Things. This method addresses the challenges of decreased detection accuracy in anomaly detection due to the heterogeneity of IoT devices and interference within IoT systems. The approach is capable of learning and capturing robust and useful features that are not significantly affected by unstable environments. Subsequently, a classifier utilizes these features to enhance the accuracy of detecting malicious IoT data. Experimental validation conducted on a real-world IoT dataset demonstrated the effectiveness of the proposed framework in improving the accuracy of detecting malicious data when compared to state-of-the-art IoT-based anomaly detection models.

Although the aforementioned methods achieve promising results in handling drift data, little attention has been paid to optimizing system performance. In real-world scenarios, IoT devices often operate under resource-constrained conditions such as limited network bandwidth, computational power, battery capacity, or storage size. Furthermore, the proposed Internet of Things concept drift methods in [17,18,19] are suitable for drift data with minor distribution changes or gradual distribution shifts. However, applying them to sudden or previously unseen drift data is challenging. Therefore, there is a need to design a lightweight and efficient detection model that adapts to various types of concept drift.

## 3. Proposed Method

**Figure 1:**
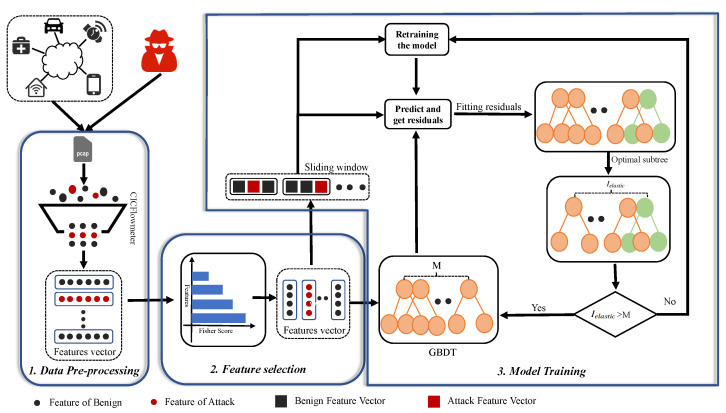
The framework of GBDT-IL.

In this section, we propose a GBDT-IL-based method for detecting IoT botnet traffic. It consists of three main components: data preprocessing, feature selection, and model training. Figure 1 shows the framework of GBDT-IL.

### 3.1. Data Pre-Processing

The captured packets are first passed through the CICFLowmeter feature extractor to obtain an over 80-dimensional feature vector. Then, the discrete and continuous feature values are transformed to range between 0 and 1 by one-hot encoding and normalization, respectively. The normalization is given by Formula (1), where $x_{min}$ and $x_{max}$ represent the minimum and maximum values of the feature, respectively.

(1) $$\begin{array}{c} x^{\prime }=\frac{x-x_{min}}{x_{max}-x_{min}} \end{array}$$

### 3.2. Feature Selection

There are many existing botnet detection systems, such as the following: based on behavioral features [20,21,22], based on honeypots [23,24,25], based on network features [26,27], and so on. However, in the IoT environment, devices in edge networks typically have limited computational resources, network bandwidth, and storage capacity. This poses significant challenges for the deployment of zombie network detection systems that require substantial computational resources [28]. Therefore, some effective methods are needed to improve the performance of detection systems. Feature selection is one of the popular methods to select the most relevant or informative subset of features from the original feature set, which can improve model performance, reduce overfitting, increase computational efficiency, and reduce the interpretability of the model [29].

In our method, we use Fisher Score to select the best features. It calculates the Fisher Score for each feature and ranks the importance of the features based on the scores. Then, irrelevant and redundant features are filtered out and important features are retained to improve model performance. Specifically, the Fisher Score evaluates significant features by intra-class feature distance and inter-class feature distance. The calculation rule is as follows: define a total of c classes $\omega_{1},\omega_{2},\cdots ,\omega_{c}$ and *n* samples in the data set. Each class contains $n_{i}$ samples. $x^{k}$ is defined as the value of a specific sample *x* at the *k*-th feature. $m_{i}^{k}$ denotes the mean of the values taken by all samples of class *i* on the *k*-th feature, and $m^{k}$ denotes the mean of the values taken by samples of all classes in the dataset on the *k*-th feature. The interclass variance $S_{B}^{k}$ and intraclass variance $S_{w}^{k}$ defined at the *k*-th feature on the dataset are given by Equation (2) and Equation (3), respectively. The Fisher Score function $J_{fisher}\left(k\right)$ is defined for the *k* features in the data set, and Formula (4) is given. It can be seen that when the interclass variance is larger and the intraclass variance is smaller, the Fisher Score is larger, and then the feature is more discriminative.

(2) $$S_{B}^{k}=\sum_{i=1}^{C}{\left(m_{i}^{k}-m_{i}\right)}^{2}$$

(3) $$S_{w}^{k}=\sum_{i=1}^{n_{k}}{\left(x_{i}^{k}-m_{i}^{k}\right)}^{2}$$

(4) $$J_{fisher}\left(k\right)=\frac{S_{B}^{k}}{\sum_{k=1}^{C}\left(n_{k}S_{w}^{k}\right)}$$

**Figure 2:**
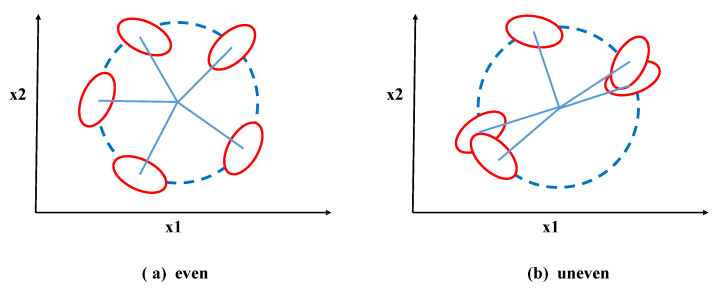
Examples of different distributions.

From Formula (2), the value of the interclass variance of the Fisher Score is obtained by re-summing the squared difference between the mean of all samples in a class under the feature and the mean of all samples in the class. However, when faced with samples on a circle with a fixed radius centered at the class centroid, as shown in Figure 2, the calculated interclass variance of the features using Formula (2) for both Figure 2a,b is the same. This clearly contradicts the actual situation. Although these samples belong to the same class centroid, and the distances from the five classes to their respective centroids are the same, the positions of the class centroids themselves are different. Therefore, the traditional Fisher Score method is unable to measure this interclass difference accurately.

**Figure 3:**
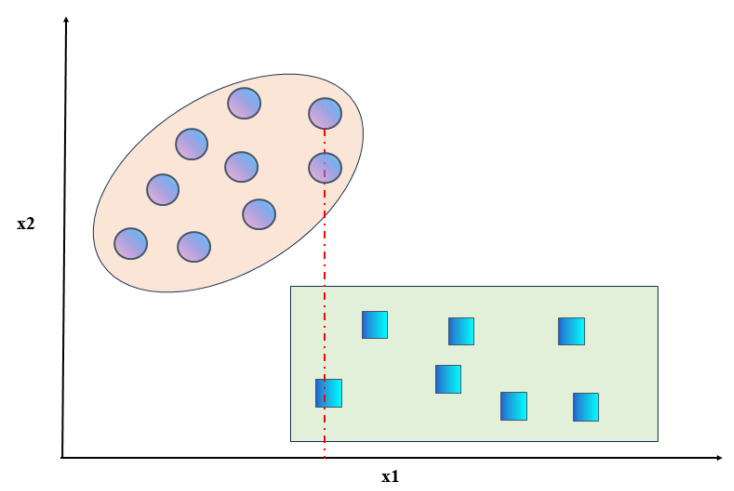
Example of overlap between classes.

To address this issue, Song et al. [30] proposed an improvement to the interclass variance formula in the Fisher Score. They modified it to be the squared difference between the average value of all samples in a certain class and the average value of samples from all other classes, as shown in Equation (5). Although this method can capture the interclass differences illustrated in Figure 2, it does not consider cases where there is an overlap in the number of samples between two classes for certain features. For example, as shown in Figure 3, when there is an overlap in a certain feature between two classes, indicated by the dashed line, the values of both classes are identical for feature x1. Therefore, taking into account the overlap among the data, we address the situation of overlapping features by introducing the cross-correlation coefficient [31]. We propose an optimized Fisher Score (NFS) method to further optimize the interclass variance. The formula for the crossover coefficient is given by Equation (6). In this equation, $n_{pq}^{\left(i\right)}$ represents the number of data points that have the same value for categories *p* and *q* under feature *i*. This value is obtained by subtracting the overlapping data to distinguish the overlapping features. If there are more overlapping data, it means that it is more difficult to distinguish the two types of data using the feature. Additionally, when there is a higher amount of overlapping data, the crossover coefficient $O_{pq}^{\left(i\right)}$ decreases, indicating a smaller interclass variance and implying that the feature poses greater difficulty in distinguishing between the classes. Therefore, combining the above two shortcomings of the existing interclass variance calculation formula, the final interclass variance is modified to Formula (7). Correspondingly, the new Fisher Score for the kth feature on the dataset is defined as $NJ_{fisher}\left(k\right)$, and its calculation is given by Equation (8).

(5) $$m=\frac{\sum_{j=1}^{5}\sum_{i=1}^{n_{j}}C_{ij}}{n}$$

(6) $$O_{pq}^{\left(i\right)}=n_{p}+n_{q}-n_{pq}^{\left(i\right)}$$

(7) $$S_{B}^{k}=\sum_{1\leq p<q\leq C}\left(\frac{n_{p}+n_{q}-n_{pq}^{\left(i\right)}}{n}\right){\left(m_{i}^{p}-m_{i}^{q}\right)}^{2}$$

(8) $$NJ_{fisher}\left(k\right)=\frac{\sum_{1\leq p<q\leq C}\left(\frac{n_{p}+n_{q}-n_{pq}^{\left(i\right)}}{n}\right){\left(m_{i}^{p}-m_{i}^{q}\right)}^{2}}{\sum_{k=1}^{C}\left(n_{k}S_{w}^{k}\right)}$$

### 3.3. Model Training

In order to efficiently detect botnet attacks in IoT, we choose the high-performance integrated learning model. Also considering the impact of concept drift data on the model, we adopt incremental learning to update the model in a timely manner. In addition, to further reduce the computational cost during model updates, we choose the GBDT model consisting of multiple regression trees. The predictions of the GBDT model are the sum of outputs from the tree ensemble, and its incremental learning can be easily achieved by adding new trees. Specifically, the GBDT model uses a portion of the data instances as the training set for training, and the remaining data instances are divided into blocks for use in subsequent iterations to fine-tune the model.

**Figure 4:**
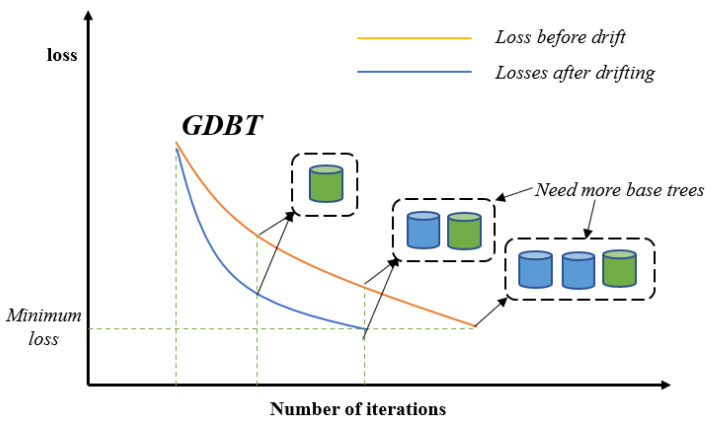
GBDT for model loss reduction.

In order to reduce the model loss in the face of drifting data, the model needs more base trees to be fitted to help the model loss reach the global optimal loss point as shown in Figure 4. Therefore, we propose the initial GBDT-IL(iGBDT-IL) algorithm. It uses a sliding window mechanism to train the GBDT model using data in the initial window and incrementally learns new data in the next window to reduce model loss due to concept drift. The exact flow is given by Algorithm 1. Specifically, a GBDT model is first trained based on the given initial data sample, and then the model is used to predict the data in the new arrival data window and calculate the residual values. Using the incremental learning approach, we utilize the residuals of the data in the new data window as a new training set to train a new decision regression tree. Subsequently, this newly trained tree is incorporated into the original GBDT model, effectively updating the model. For the binary classification problem, the initial training window and the new sliding window are considered as $D_{chunk_{ini}}=\left\{{\left(x_{i},y_{i}\right)}_{i=1}^{chunk_{ini}}\right\}$ and $D_{chunk_{slide}}=\left\{{\left(x_{i},y_{i}\right)}_{i=1}^{chunk_{slide}}\right\}$, respectively, where the formula for predicting the residual values is shown in Equations (2)–(6), and then setting the learning rate to λ, the number of incremental trees is assumed to be *L*. A new regression tree is generated by fitting with {${\left(x_{i},\lambda r_{i(M+L)}\right)}_{i=1}^{chunk_{slide}}$}, and the model is updated by Formula (9).

(9) $$F_{M+L}\left(x\right)=F_{M+L-1}\left(x\right)+h_{M+L}\left(x\right)$$

This is easier to compute than the traditional GBDT learning because a fixed learning rate is set. The same algorithm is applied to the newly arrived sliding window, and the GBDT model is progressively adjusted, so that the model can gradually adapt to conceptual drift. However, the initial GBDT-IL algorithm only adapts the model to the new data distribution by simple incremental learning when too many classification subtrees in the model may cause overfitting problems in the model. For example, if an attack traffic has been trained and accurately classified by the model, and the attack traffic is encountered again without conceptual drift, the attack traffic will be trained again if the model is updated using the method described in initial GBDT-IL, and this redundant step in the training process may lead to overfitting. To address this, we tackle the issue by pruning the regression trees in the GBDT model. Meanwhile, the initial GBDT-IL algorithm does not take into account the detection of conceptual drift, although it can adapt to conceptually drifted data. Therefore, we draw inspiration from drift detection algorithms based on the classification model performance [15,32,33,34]. We devise a concept drift detection algorithm called GBDT-IL. Specifically, on the new sliding window data, a decision regression tree is constructed and directly added to the GBDT model. However, to update the entire model rather than just adding a new tree, further processing is required. This is because the GBDT model’s prediction results are obtained by sequentially summing the predicted values of each decision regression tree. In order to update the model effectively, we need to identify the first m trees that, when combined, result in a cumulative prediction closest to the actual sample values. The fine-tuning of the decision regression trees within the GBDT is performed, retaining only the first m trees to prevent the overfitting of the model. The learning process of GBDT-IL is shown in Algorithm 2. Here are some key implementation details of the algorithm: the prediction of the GBDT model is the sum of the predictions of each tree within the model, and the prediction of the *m*-th tree on a given feature vector *x* is denoted as ${\hat{y}}_{m}=h_{m}\left(x\right)$. Then, the prediction of all GBDT trees for *x* is ${\left\{{\hat{y}}_{m}\right\}}_{m=1}^{M}$. The final prediction on the *m*-th tree is the sum of the predictions from the first tree to the *m*-th tree, as shown in Equation (10).

(10) $$F_{m}\left(x\right)=\sum_{i=1}^{m}h_{i}\left(x\right)+\bar{y}$$

$\bar{y}$ represents the average value of the labels, which corresponds to the initial predictions of the GBDT model. The first regression tree $h_{0}$ is trained in $D_{chunk_{ini}}$ with the mean of the labels $\bar{y}$ as the initial target variable, and the residuals are computed by $R_{0}=y-\bar{y}$. The residual vector of the m-th tree in the data block of the sliding window can be represented by Equation (11).

(11) $$R_{m}=y-F_{m}\left(x\right)=y-\bar{y}-\sum_{i=1}^{m}h_{i}\left(x\right)$$

To improve operational efficiency, the residuals are stored in a matrix of size *M*. Then, the best-performing sublist of the tree can be found by finding the minimum average absolute value (MA) residuals.

(12) $$I_{elastic}=argminMA\left(R_{m}\right)$$

$I_{elastic}$ is the GBDT tree index. At this point, $GBDT=\{h_{0}\left(x\right),\cdots ,h_{I_{elastic}}\left(x\right)\}$ represents the optimal subtree list of GBDT, while the redundant subtrees $\left\{h_{I_{elastic+1}}\left(x\right),\cdots ,h_{M}\left(x\right)\right\}$ are discarded. If $I_{elastic}$ is less than a predefined threshold, a significant conceptual drift is considered, and the new GBDT is retrained based on the new data. To mitigate the issue of overfitting during the retraining of the GBDT model, we perform pruning on the base trees of the GBDT. At the beginning of the data stream, a GBDT with *M* trees is constructed on the initial training window $D_{chunk_{ini}}$. Then, for each new chunk of data predicted, pruning and drift detection are performed, and the pruned GBDT is fitted with L new tree increments on $D_{chunk_{slide}}$.

In the GBDT-IL algorithm, the initial number of trees M is used as the drift threshold. If $I_{elastic}<M$, it triggers the retraining process. Additionally, as the model adapts to new concepts through incremental learning, the number of subtrees in the GBDT model is expected to increase with the progression of the data stream. If it is observed that the total number of trees in the model, after pruning, is still less than the number of trees trained on the initial window, it indicates that the existing model cannot adapt to the new data in the data stream. In such cases, the GBDT model is retrained on the new sliding window, and the drift threshold is updated to *M*, representing the number of trees in the retrained GBDT model.
**Algorithm 1** initial GBDT-IL**Input:** Initial training window $D_{chunk_{ini}}$, Sliding window $D_{chunk_{slide}}$
**Output:** Incremental GBDT model $F_{M+L}\left(x\right)$
 1: Initialization
 2: Train a GBDT model in the initial training block M $F_{M}\left(x\right)$
 3: **for**
l=1 to *L* **do**
 4:    Calculate the pseudo-residuals in a new slider using Equations (2)–(5)
 5:    Fitting the data in the new slider to generate a new regression tree
 6:    Update the model by Equations (3)–(5)
 7: **end for**
 8: return $F_{M+L}\left(x\right)$


**Algorithm 2** GBDT-IL**Input:** Trained GBDT model $F_{M}\left(x\right)$, Slider size $D_{chunk_{slide}}$
**Output:** GBDT model after pruning, $F_{M^{\prime }}\left(x\right)$
 1: Testing the GBDT model in a slider $F_{M}\left(x\right)$
 2: Calculate each tree residual value based on the model output
 3: Find the tree that minimizes the average absolute value of the residuals, and the number of trees is denoted as $m^{\prime }$
 4: **if**
m<M **then**
 5:    Retrain the GBDT model $F_{M}\left(x\right)$ on a sliding block
 6:    Return the model after retraining
 7: **else**
 8:    Pruning of the model
 9:    Return the model after pruning
 10: **end if**


## 4. Experimental Evaluation and Results

### 4.1. Experimental Environment

We evaluated our proposed approach on a device with an Intel(R) Core(TM) i5-4590 CPU @3.30GHZ, NVIDIA GeForce GTX 1050 GPU, 16G RAM and Windows 11. The python 3.8.8, WireShark 3.6.6, CICflowmeter V4.0, node-red 1.3, and MQTT Broker emq 3.1.2 software environments were also used.

### 4.2. Dataset Description

In this study, we mainly utilized the N-BaIoT [35], BoT-IoT [36], MedBIoT [9], and MQTTSet datasets [37] to test our proposed approach, and the specific information of the datasets is shown in Table 1.

The N-BaIoT dataset was generated by Yair Meidan et al. [35] from two networks: one is an IP camera video surveillance network where eight different types of attacks were launched to affect the availability and integrity of the video uplink; second, an IoT network consisting of personal computers and real IoT devices, including doorbells, security cameras, and webcams, where a computer was infected with malware from the Mirai botnet. The attack types include OS Scan, Fuzzing, Video injection, ARP MITM, Active Wiretap, SSDP flood, SYN DoS, SSL Renegotiation, and Mirai botnet. This dataset construction approach is more aligned to the realistic IoT environment.

The BoT-IoT dataset was constructed by Nour Moustafa et al. [36] through two main efforts to simulate the IoT environment and collect malicious traffic in IoT scenarios. Firstly, to capture benign IoT device traffic, they used Raspberry Pi as a virtual IoT sensor device and developed programs to simulate IoT devices such as temperature, humidity, and ambient light sensors. The network traffic was captured using Tcpdump. Secondly, the malicious traffic was obtained from the intrusion traffic dataset UNSW-UB15, and the two types of traffic were combined to create the final dataset. Overall, the captured benign IoT device traffic in this approach is virtual, and the attack traffic does not reflect the contagious nature of a botnet.

The MedBIoT dataset was collected by Alejandro Guerra-Manzanares et al. [9] by deploying three botnet malware from the botnet infection, propagation, and communication phases (Mirai, BashLite, and Torii). And the dataset contains a total of 83 virtual and real IoT devices, filling the gap of a lack of traffic data sources for medium-sized IoT environments by providing a new dataset of network data collected from medium-sized IoT network architectures.

The MQTTSet dataset was generated by Ivan Vaccari et al. [37] by using IoT-Flock to simulate benign and attack traffic from IoT devices based on the MQTT protocol, which involves MQTT attack traffic including Flooding Denial of Service, MQTT Publish Flood, SlowITe, Malformed Data, and Brute Force Authentication.

In order to evaluate the improved Fisher Score, four IoT botnet datasets were partially censored, in which 15 features such as protocol type, source ip address, destination ip address, data classification, and 18 other features that cannot be used for training were removed from the BoT-IoT dataset; 7 features such as data label and data classification were removed from the MQTTSet dataset. The MQTTSet dataset removes 7 features that are not effective for model training. The basic information of the four deleted datasets is shown in Table 2.

In the four datasets, none of the datasets exhibit conceptual drift. Inspired by the literature [38,39,40], we used the four IoT data to artificially construct a dataset that generates data drift based on the relevant conceptual drift definitions. The dataset combines various types of attacks, including botnet TCP_DoS attacks, DDoS attacks, Theft attacks, and DoS attack traffic generated using the IoT_Flock software (https://github.com/ThingzDefense/IoT-Flock) from the BoT-IoT dataset. Additionally, the benign traffic is sourced from the benign traffic in N_BaIoT and the virtual IoT device traffic generated using the Node Red Software Version 3.10 with the MQTT 5.0 protocol The detailed traffic composition of the dataset is shown in Table 3.

### 4.3. Evaluation Criteria

In order to accurately evaluate the merits of detection models, this study used two evaluation criteria commonly used today: accuracy and false alarm rate (FPR).

Accuracy: the proportion of correctly predicted samples to all samples, which reflects the overall ability of the detection system to distinguish all flows in the controlled monitoring area, as shown in Formula (13).

(13) $$\begin{array}{c} Accuracy=\frac{TP+TN}{TP+FN+FP+TN} \end{array}$$

False alarm rate: the proportion of the predicted wrong positive samples to all positive samples, also called false identification rate. The false alarm rate reflects the ability of the detection system to distinguish the benign traffic in the monitored area; the specific formula is shown in Formula (14).

(14) $$\begin{array}{c} FPR=\frac{FP}{FP+TN} \end{array}$$

TP is the number of samples classified as positive cases, TN is the number of samples classified as negative cases, FP is the number of samples classified as positive cases, and FN is the number of samples classified as positive cases.

### 4.4. Concept Drift Design

In order to test the performance of the anti-concept drift algorithm proposed in this paper, we used the dataset in Table 2 to design a subset of data with three different ways of concept drift according to the type of concept drift and combined it with the reality of IoT botnets.

Concept drift type I simulates the scenario where the attack itself undergoes changes over time. Specifically, it includes the same type of attack from different botnet families, as well as changes in attack methods for the same attack type over time. To address these two scenarios, we designed two concept drift data subsets as shown in Table 4, where number represents the amount of data for the selected class category.

Concept drift type II simulates the scenario where the attack methods change, introducing new attack types that were not previously trained by the model. Specifically, it represents the situation where a particular botnet suddenly introduces new attack methods different from its previous ones. To address this scenario, we designed concept drift data subsets as shown in Table 5.

The concept drift III presents a more complex realistic environment in which there are both concept drift types I and II, and at the same time, the distribution of various attack traffic varies. In this case, in order to test the resistance of our model to concept drift, we designed a subset of data where concept drift occurs as shown in Table 6.

### 4.5. Parameter Setting

Considering the complexity of concept drift type 3, which involves 100,000 data instances, and aiming to balance the model training time and classification accuracy, we set the sliding window size to 500 and the initial training set to 1000 instances. In addition, the GBDT parameters used in the experiments and all the parameters of GBDT-IL are shown in Table 7.

### 4.6. Baseline Setting

To evaluate the improved-Fisher-Score feature selection algorithm, we compared it with the following feature selection methods: the traditional Fisher Score method [16], the DFS method proposed by Juan-Ying Xie et al. [41], and the Weighted Feature Selection (WFS) method proposed by Zhao et al. [42]. Xie proposed the DFS algorithm by utilizing the improved F-score as a filter evaluation criterion and SVM as a wrapper evaluation method, the approach aims to remove irrelevant redundant features and obtain an optimal feature subset. WFS involves the block-wise processing of face images to extract local binary pattern (LBP) features within each block, which are then concatenated to form a global feature vector. Subsequently, a support vector machine (SVM) classifier is trained on these feature vectors for gender recognition. In addition, to validate the effectiveness and feasibility of the GBDT-IL algorithm, we selected Learn++.NSE [43], OnlineAUE [44], HDDM-W-Test [45], and HDDM-A-Test [45] as benchmark algorithms for comparison.

### 4.7. Results and Analysis

Since this paper is about a botnet detection model, all attack traffic is defined as a malicious traffic in this paper, that is, the algorithm is a binary classification algorithm that only distinguishes benign traffic from malicious traffic regardless of the number of traffic categories in the data set.

#### 4.7.1. Feature Dimensionality Reduction

**Figure 5:**
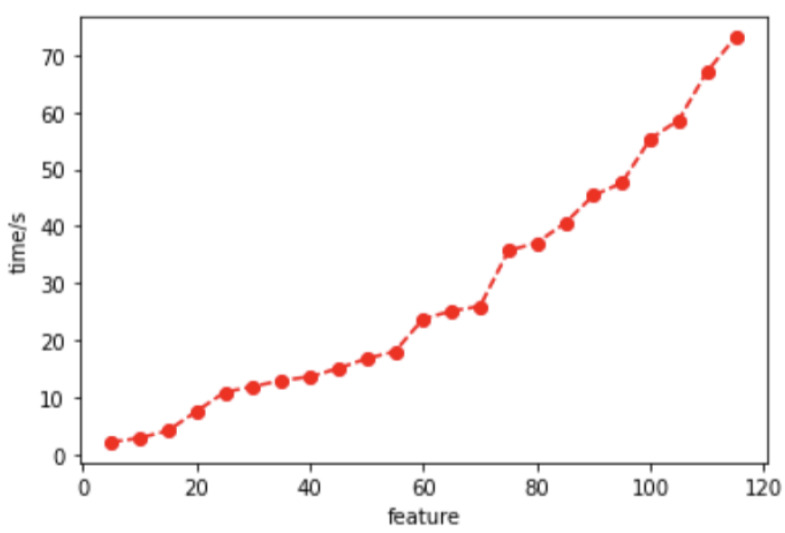
Relationship between the number of features and model training time consumed.

In order to illustrate the necessity of feature dimensionality reduction, the relationship between the number of features and the model training’s elapsed time was tested on the N-BaIoT dataset, as shown in Figure 5. It can be observed that the training time in the decision tree algorithm exhibits a linear increase with the number of features, further emphasizing the necessity of feature dimensionality reduction in resource-limited IoT environments.

We conducted multiple experiments using four feature dimensionality reduction algorithms on the four IoT datasets shown in Table 2. Table 8 summarizes the highest accuracy achieved and the number of features selected for each algorithm. From the table, it is evident that, except for the MQTTSet dataset, the other three datasets achieved a higher classification accuracy when using a subset of features obtained through feature dimensionality reduction compared to using all features for model training. For instance, in the N-BaIoT dataset, the improved Fisher Score feature dimensionality reduction method achieved a higher accuracy with only 55 features used for model training compared to using all 115 features in the dataset. According to Figure 5, it can be observed that the training time for the decision tree model with 55 features is less than half of the training time with 115 features, resulting in significant savings in system resource consumption. Additionally, all four Fisher Score methods showed improvements in classification accuracy and a reduction in the number of features across the four datasets. However, when comparing these four methods, the proposed improved Fisher Score method consistently performed the best in terms of achieving the highest accuracy among the four datasets.

**Figure 6:**
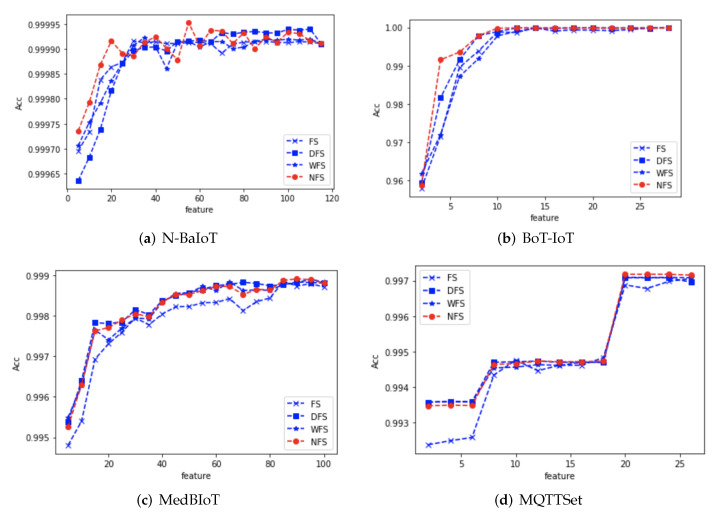
Relationship between the number and accuracy of features for the four datasets.

In addition, to more visually represent the relationship between the accuracy of the detection models and the number of features for the four data sets, the accuracy of the detection models versus the number of features is plotted as shown in Figure 6a–d.

We analyze the relationship between accuracy and the number of features for the four detection models based on the respective graphs:

N-BaIoT dataset: the classification accuracy of the detection model increased gradually with the increase in the number of features when the features ranked by the four Fisher Score methods were used for model training, among which the proposed NFS reached the peak accuracy when the number of features was 55, which was also not reached by the other three feature selection methods. Simultaneously, we observed that FS and NFS achieved a relatively high accuracy within the first 20 features, while DFS lagged slightly behind the other three methods. This indicates that the N-BaIoT data can be well classified with a limited number of features. Therefore, DFS needs to eliminate a significant amount of redundant features, resulting in a slightly lower accuracy within the first 20 features.

BoT-IoT dataset: when using the feature ranking provided by the four Fisher Score methods for model training, the classification accuracy of the detection models gradually increased with the increasing number of features. Among them, the proposed NFS in this paper reached its peak accuracy at 12 features, which was only achieved by DFS. Additionally, considering the overall performance of accuracy across different numbers of features, the proposed feature selection algorithm in this paper was also superior. Within the first six features, each additional feature had a significant impact on the accuracy of all four methods. This suggests that these features might be crucial for classification. It is noteworthy that the fifth and sixth features resulted in a substantial difference in classification outcomes between NFS and FS. This can be attributed to the considerable inter-class differences present in the dataset.

MedBIoT dataset: the classification accuracy of the detection models gradually increased with the increasing number of features. Among them, the proposed NFS in this paper reached its peak accuracy at 12 features, which was also not achieved by the other three feature selection methods. Considering the overall classification accuracy across different numbers of features, only the traditional Fisher Score feature selection method performed slightly worse than the other three methods. DFS, WFS, and the proposed NFS showed a similar performance overall. This is because the subsequent features in the MedBIoT dataset exhibited feature overlap. The dataset demonstrated effective classification within the first nine features, and the addition of further features had a minimal impact on classification.

MQTTSet dataset:The classification accuracy of the detection model generally increased with the increasing number of features, but the distribution of accuracy in this dataset was discontinuous. Additionally, the highest accuracy was achieved with a feature count close to the total number of features. In the case of the proposed NFS, the peak accuracy was reached at a feature count of 20. This suggests that the dataset might involve combined features that influence classification, warranting further exploration.

In summary, the experimental results across the four datasets reveal that FS performs well on simple and easily classifiable datasets, while slightly trailing behind NFS on more complex datasets due to its insensitivity to inter-class differences. DFS, relying on redundant feature elimination, may lag behind NFS on simple datasets as it needs to remove excessive redundant features, but its performance is comparable on other datasets. WFS, designed for image domains, demonstrates results close to NFS on all four datasets. Therefore, exploring how to better integrate WFS for traffic feature selection is a worthwhile avenue for investigation.

#### 4.7.2. Concept Drift Detection

To further simulate real zombie network attacks, we designed two potential scenarios. The first scenario (T1) involves a periodic occurrence of a specific type of attack in both drifting and non-drifting normal data. For example, a Mirai_Scan attack appears approximately every five sliding windows. The second scenario (T2) entails a mixture of drifting and non-drifting normal data, randomly appearing together within a single sliding window. Next, we proceed to a detailed analysis of the performance of the proposed method in various drift scenarios outlined in this paper.

**Figure 7:**
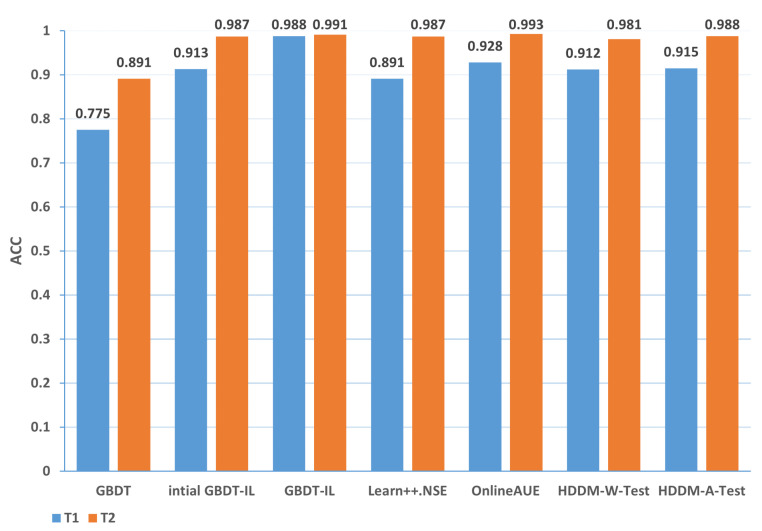
Comparison of accuracy of different algorithms under concept drift type I.

**Figure 8:**
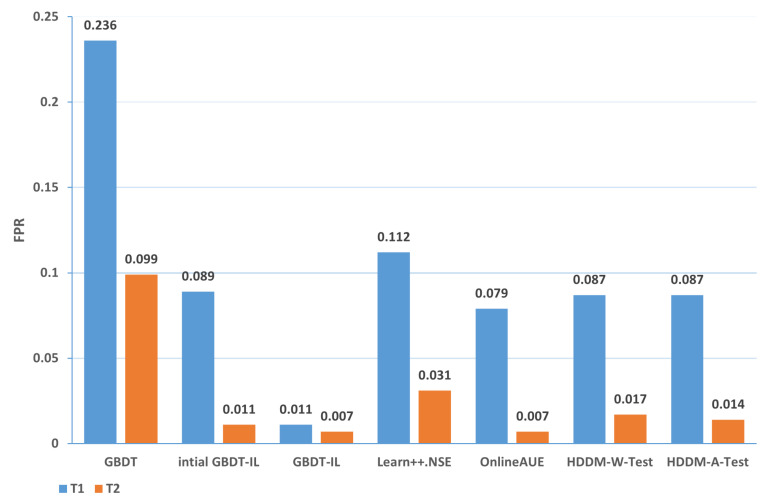
Comparison of false positive rates of different algorithms under concept drift type I.

**Figure 9:**
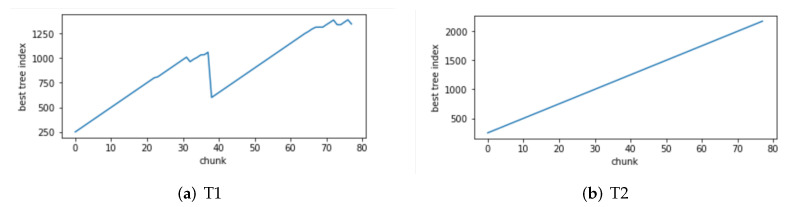
Variation of the number of GBDT-IL trees with sliding window at concept drift type I.

Change of the attack itself:The comparison of different algorithms in terms of accuracy for concept drift types T1 and T2 is shown in Figure 7, while the false positive rates are shown in Figure 8. It can be observed that the GBDT-IL algorithm achieves the highest classification accuracy for concept drift type T1, while its accuracy is slightly lower than the OnlineAUE algorithm for concept drift type T2. In both cases of concept drift type T1, GBDT-IL exhibits the lowest false positive rates. Additionally, it is observed that although the initial GBDT-IL has a lower accuracy in T1, its accuracy is very close to GBDT-IL in T2. To facilitate analysis, the relationship between the number of trees trained in the GBDT-IL model and the size of the sliding window is plotted in Figure 9 for T2. It can be observed that the number of trees increases continuously with the size of the sliding window, indicating incremental learning. In this scenario, GBDT-IL and the incremental learning model, initial GBDT-IL, achieve a similar accuracy. However, in the case of concept drift type T1, it can be seen from the graph that GBDT-IL clearly detects the drift at around a sliding window size of 40 and performs the process of pruning redundant trees at the concept drift point. Therefore, in such a scenario, the performance of initial GBDT-IL is significantly inferior to GBDT-IL.

**Figure 10:**
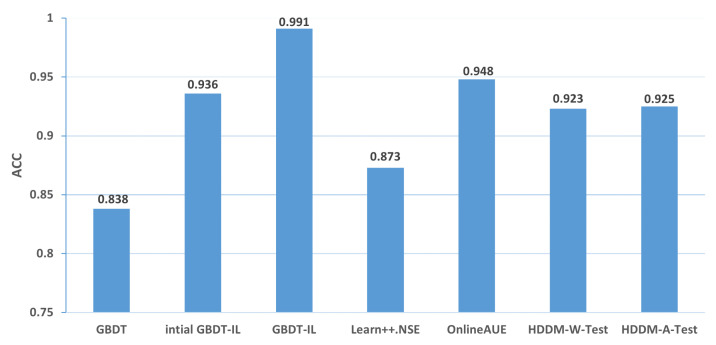
Comparison of accuracy of different algorithms under concept drift type II.

**Figure 11:**
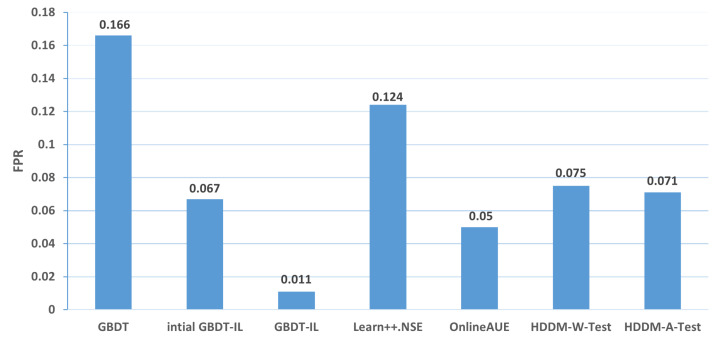
Comparison of false positive rates of different algorithms under concept drift type II.

**Figure 12:**
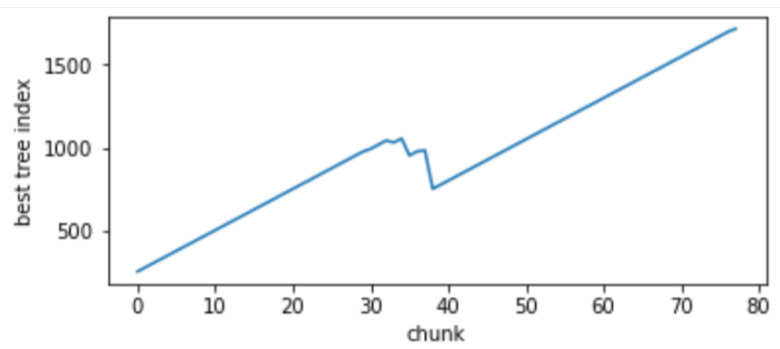
Variation of the number of GBDT-IL trees under concept drift type II with sliding window.

Changes in the mode of attack: Comparisons of the accuracy and false alarm rates of different algorithms when concept drift type II occurs are shown in Figure 10 and Figure 11, respectively. It can be seen that the classification accuracy of the GBDT-IL algorithm is the highest when concept drift type II occurs, and the relationship between the number of trees and the sliding window during training is shown in Figure 12.

**Figure 13:**
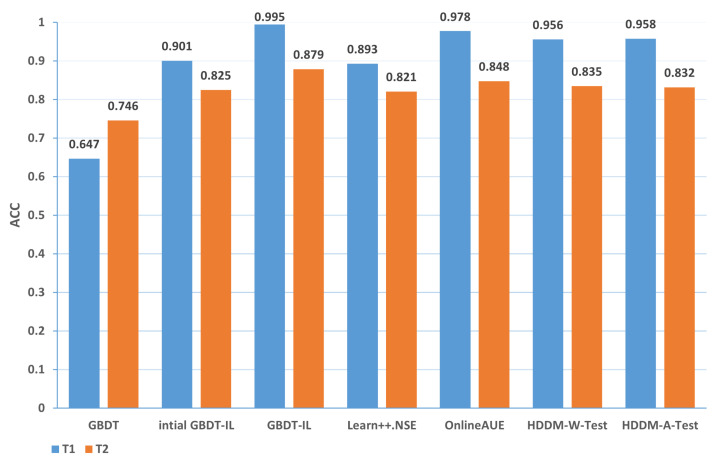
Comparison of accuracy of different algorithms under concept drift type III.

**Figure 14:**
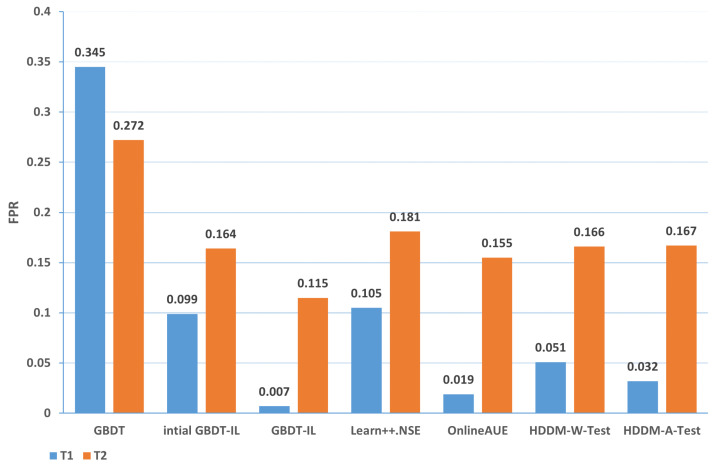
Comparison of false positive rates of different algorithms under concept drift type III.

Mixing the above two cases and the conceptual drift of data distribution changes: the comparison of different algorithms in terms of accuracy and false positive rates for concept drift types T1 and T2 of type 3 are shown in Figure 13 and Figure 14, respectively. It can be observed that the GBDT-IL algorithm achieves the highest classification accuracy and lowest false positive rates for both concept drift types T1 and T2 of type 3. This indicates that GBDT-IL is capable of adapting to different types of concept drift and performs remarkably well.

**Figure 15:**
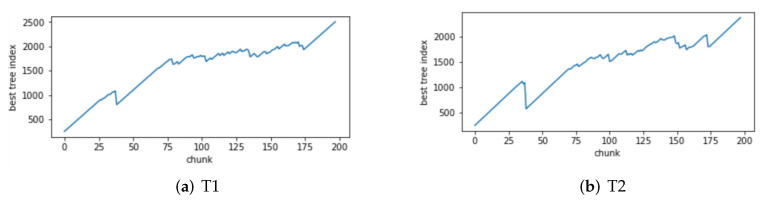
Variation of the number of GBDT-IL trees under concept drift type III with sliding window.

The relationship between the number of trees in GBDT-IL during training and the sliding window for concept drift type 3 is illustrated in Figure 15. As shown in Figure 15a, during drift point 1 (from concept C1 to C2), there is previously unseen attack traffic in the data, leading to a noticeable process of pruning redundant trees and retraining. Figure 15b demonstrates the response of the model to drift point 4 (from concept C4 to C5) where the attack method changes from Scan attack in the Mirai botnet to Scan attack in the Gafgyt botnet. The model adapts by training new trees to handle the emergence of the new attack type. These figures visually depict the ability of the proposed GBDT-IL approach to handle different types of concept drift and its outstanding performance.

In summary, the accuracy of different algorithms under various types of drift is presented in Table 9, while the false positive rate is shown in Table 10. In the tables, 1-T1 represents the first subset of data for concept drift type 1, which consists of attacks from different zombie network families of the same type. The same notation is used for 1-T2, 2, 3-T1, and 3-T2. The rankings represent the average ranking across the five concept drift types. Based on the analysis, the following conclusions can be drawn:

(1) From the perspective of accuracy and false alarm rate, the improved anti-conceptual drift algorithm proposed in this paper has a higher accuracy and lower false alarm rate compared with the other six algorithms, and our proposed algorithm is ranked first in terms of five different types of conceptual drift.

(2) Under different types of drift, intial GBDT-IL significantly improved the performance of accuracy and false alarm rate compared with the original GBDT model, and the accuracy can be improved by up to 25.4% and at least 7.9%, which shows that incremental learning is useful for detecting conceptual drift.

(3) Under five types of concept drift, for four types, 1-T1, 2, 3-T1, and 3-T2, the classification performance of the algorithm GBDT-IL with the addition of the pruning GBDT tree operation is significantly improved compared with the intial GBDT-IL algorithm without this operation, and the accuracy is improved by up to 9.4% and the false alarm rate is reduced by up to 9.2 %, illustrating the effectiveness of the further optimization of the model proposed in this paper.

(4) Combined with analysis (3), we can see that the performance of GBDT-IL is not significantly improved compared with the intial GBDT-IL model at drift type 1-T2. Combined with the experimental results in Figure 9 we can find that the GBDT-IL model at 1-T2 does not trigger the process of pruning the GBDT tree or model retraining, but only keeps increasing the number of GBDT subtrees. In this case, the model performance is not significantly different from the pure incremental learning model of initial GBDT-IL.

(5) In an experimental environment with relatively low data traffic, once the distribution of data changes, the classification accuracy of the model can drop to at most 64.7% when using the traditional machine learning model GBDT. In the actual IoT environment, the data traffic is much larger, the types of data are more diverse, and the distribution of data is more variable, thus also fully illustrating the need to add an anti-conceptual drift algorithm to the botnet traffic detection model used in the real environment.

## 5. Conclusions and Future Work

In this paper, we propose an incremental learning model based on GBDT, GBDT-IL, which adapts to the occurrence of drifting data by incrementally learning to construct new GBDT subtrees. In addition, we propose a feature selection method with improves the Fisher Score to perform feature dimensionality reduction on the dataset features, which allows the model to reduce the model training speed while maintaining the detection rate. To evaluate the performance of the model, we tested our approach on four commonly used IoT datasets as well as their constructed drift datasets. The experimental results show that the improved Fisher Score’s feature selection method outperforms the existing feature selection methods, significantly reducing the training time of the model. In addition, GBDT-IL is able to improve the model accuracy by more than 20% compared to traditional machine learning algorithms, and it also performs better than existing concept drift resistant algorithms.

We have made some improvements to the existing botnet detection model for the feature reduction part and the anti-concept drift part, both of which have good experimental results, but these methods still have shortcomings to be improved. When we filter features, we simply consider the score, and the features with a high score are filtered and used as a priority. For example, the four features with the highest to lowest Fisher Score are x1,x2,x3,x4. If two features are used for model training, x1 and x2 are selected for model training according to the screening method proposed in this paper, but the training effect may not be better than x1 and x3 for model training. The method can be further enhanced in the future by considering the problem of feature combination optimization. In addition, the proposed algorithm utilizes the residual difference between the model prediction results and the existing results to update the model, and it is a supervised learning algorithm. However, in real production, the existing traffic may not be judged as benign or malicious by a simple method, so how to use the proposed supervised learning algorithm in a realistic environment remains to be pondered.

## Figures and Tables

**Table 1:** Comparison of the four data sets.

Dataset	Botnet	Real/Virtual Device	Time	Description
N-BaIoT	Mirai&BashLite	real	2018	More relevant to real IoT environments
BoT-IoT	Virtual	Virtual	2018	Multiple protocol types and attack traffic samples
MedBIoT	Mirai&BashLite	real + Virtual	2020	Simulated medium-sized IoT network
MQTTSet	Virtual	Virtual	2020	Focused on the MQTT protocol

**Table 2:** Basic information about the dataset.

Dataset	Samples	Types	Data Set Features	Model Training Features
N-BaIoT	7,062,606	10	115	115
BoT-IoT	73,370,443	7	46	28
MedBIoT	17,845,567	4	100	100
MQTTSet	156,261	6	34	27

**Table 3:** Data set composition.

Dataset	Composition
IoT botnet traffic	BoT-IoT-Malware-TCP_Dos
	BoT-IoT-Malware-TCP_DDos
	BoT-IoT-Malware-Theft
	Dos attack traffic generated by IoT-Flock
	N_BaIoT_Mirai
	N_BaIoT_gafgyt
Normal Traffic	N_BaIoT_Benign
	Virtual traffic based on MQTT protocol

**Table 4:** Conceptual drift type I specific experimental flow.

Concept Type	Flow Type (Number)	
The same type of attack for different botnet families		
C1	Benign (10,000)	Mirai_Scan (10,000)
C2	Benign (10,000)	gafgyt_Scan (10,000)
Same attack pattern over time		
C1	Benign (10,000)	BoT-IoT_Dos (10,000)
C2	Benign (10,000)	IoT-Flock_Dos (10,000)

**Table 5:** Conceptual drift type II specific experimental flow.

Concept Type	Flow Type (Number)	
C1	Benign (10,000)	Mirai_Scan (10,000)
C2	Benign (10,000)	Mirai_Ack (10,000)

**Table 6:** Concept drift type III specific experimental flow.

Concept Type	Flow Type (Number)			
Type I				
C1	Benign (10,000)	Mirai_Scan (10,000)	Mirai_Ack (0)	Mirai_Syn (0)
C2	Benign (10,000)	Mirai_Scan (10,000)	Mirai_Ack (10,000)	Mirai_Syn (2000)
C3	Benign (10,000)	Mirai_Scan (2000)	Mirai_Ack (2000)	Mirai_Syn (10,000)
C4	Benign (0)	Mirai_Scan (4000)	Mirai_Ack (4000)	Mirai_Syn (4000)
C5	Benign (0)	gafgyt_Scan (4000)	Mirai_Ack (4000)	Mirai_Syn (4000)
Type II				
C1	Benign (10,000)	Bot-IoT_Dos (10,000)	Bot-IoT_DDos (0)	Bot-IoT_Scan (0)
C2	Benign (10,000)	Bot-IoT_Dos (10,000)	Bot-IoT_DDos (10,000)	Bot-IoT_Scan (2000)
C3	Benign (10,000)	Bot-IoT_Dos (2000)	Bot-IoT_DDos (2000)	Bot-IoT_Scan (10,000)
C4	Benign (0)	Bot-IoT_Dos (4000)	Bot-IoT_DDos (4000)	Bot-IoT_Scan (4000)
C5	Benign (0)	IoT-Flock_Dos (4000)	Bot-IoT_DDos (4000)	Bot-IoT_Scan (4000)

**Table 7:** Model parameter setting.

GBDT		GBDT-IL	
**Parameter**	**Value**	**Parameter**	**Value**
max_iter	250	ini_train_size	1000
sample_rate	0.8	win_size	500
learn_rate	0.01	max_tree	10,000
max_depth	10	num_inc_tree	25
min_sample_leaf	5		

**Table 8:** Accuracy of different algorithms on four data sets and their selected number of features.

Dataset		Original	FS	DFS	WFS	NFS
N_BaIoT	accuracy feature	0.999911 115	0.999916 30	0.999940 110	0.999923 35	0.999953 55
BoT-IoT	accuracy feature	0.999867 28	0.999871 14	0.999876 12	0.999870 16	0.999876 12
MedBIoT	accuracy feature	0.998813 100	0.998835 80	0.998906 90	0.999870 95	0.998915 90
MQTTSet	accuracy feature	0.997173 27	0.997056 26	0.997078 12	0.997098 16	0.997178 20

**Table 9:** Accuracy of different algorithms under different types of concept drift.

	GBDT	iGBDT-IL	GBDT-IL	Learn++	OnlineAUE	HDDM-W	HDDM-A
1-T1	0.775	0.913	0.988	0.891	0.928	0.912	0.915
1-T2	0.891	0.987	0.991	0.978	0.993	0.981	0.988
2	0.838	0.936	0.991	0.873	0.948	0.923	0.925
3-T1	0.647	0.901	0.995	0.893	0.978	0.956	0.958
3-T2	0.746	0.825	0.879	0.821	0.848	0.835	0.832

**Table 10:** False alarm rate of different algorithms under different types of concept drift.

	GBDT	iGBDT-IL	GBDT-IL	Learn++	OnlineAUE	HDDM-W	HDDM-A
1-T1	0.236	0.089	0.011	0.112	0.079	0.087	0.087
1-T2	0.099	0.011	0.007	0.032	0.007	0.017	0.014
2	0.166	0.067	0.011	0.124	0.05	0.075	0.071
3-T1	0.345	0.099	0.007	0.105	0.019	0.051	0.032
3-T2	0.272	0.164	0.115	0.181	0.155	0.166	0.167
Ranking	7	3.8	1	6	1.8	4.2	3.8

## Data Availability

The data in this study were derived from the following resources available in the public domain: [N-BaIoT at https://www.kaggle.com/datasets/mkashifn/nbaiot-dataset], [BoT-IoT at https://research.unsw.edu.au/projects/bot-iot-dataset], [MedBIoT at https://cs.taltech.ee/research/data/medbiot/], [MQTTSet at https://www.kaggle.com/datasets/cnrieiit/mqttset].
